# Methyl-cantharidimide suppresses cyclin-dependent kinase 1 (CDK1) and induces oxeiptosis in liver cancer

**DOI:** 10.1186/s13046-026-03669-8

**Published:** 2026-03-02

**Authors:** Yi-Dong Li, Xiang Chen, Xing-Duo Dong, Zhiyong Ding, Zhe-Sheng Chen, Shuo Fang

**Affiliations:** 1 Department of Pharmaceutical Sciences, College of Pharmacy and Health Sciences, St. John’s University, Queens, NY 11439 USA; 2 Institute for Personalized Cancer Care, Fynn Biotechnologies Ltd., Jinan City, Shandong 250101 PR China; 3 Department of Oncology, The Seventh Affiliated Hospital, Sun Yat-sen University, Shenzhen, Guangdong 518107 PR China

**Keywords:** Methyl-cantharidimide (MCA), Liver cancer, CDK1, Oxeiptosis

## Abstract

**Background:**

Methyl-cantharidimide (MCA), a cantharidin (CTD) analog, has been approved in China for the clinical treatment of liver cancer. Previous studies have shown that MCA can induce both apoptosis and cell cycle arrest in cancer cells. However, the precise molecular mechanisms underlying its anticancer activity remain largely unclear.

**Methods:**

The effects of MCA were evaluated in liver cancer cell lines using colony formation assays. Bioinformatic analysis was performed to explore molecular mechanisms, with validation via immunoblotting and immunofluorescence. Reactive oxygen species (ROS) production was measured to assess oxidative stress induction. A 3D multicellular tumor spheroid (MCTS) model was used to mimic tumor-like in vitro conditions, and a HepG2 xenograft mouse model was employed to investigate in vivo efficacy.

**Results:**

MCA significantly inhibited colony formation in liver cancer cells. Bioinformatic profiling suggested that downregulation of cyclin-dependent kinase 1 (CDK1) may mediate MCA-induced cell cycle arrest, which was confirmed by suppressed CDK1 expression in both immunoblotting and immunofluorescence assays. MCA also increased intracellular ROS levels. Further analyses revealed that MCA-induced apoptosis occurred through a ROS-dependent oxeiptosis pathway, rather than the conventional caspase-dependent route. In the MCTS model, MCA maintained its cytotoxicity, demonstrating efficacy under more physiologically relevant conditions. In the in vivo xenograft mouse model, MCA treatment led to a significant reduction in tumor size.

**Conclusions:**

MCA exerts anticancer effects in liver cancer cells by inducing cell cycle arrest via CDK1 downregulation and promoting apoptosis through a ROS-dependent oxeiptosis pathway. These findings provide mechanistic insight into MCA’s antitumor activity and support its potential as a therapeutic agent for liver cancer.

**Supplementary Information:**

The online version contains supplementary material available at 10.1186/s13046-026-03669-8.

## Introduction

Cancer is a leading global health issue and ranks as the second most common cause of death in the United States. In the United States, 2023 projections anticipate approximately 1.96 million new cancer diagnoses and about 609,820 deaths attributable to cancer [1]. Among all cancer types, liver cancer ranks seventh in mortality [2]. Liver cancer development is commonly associated with several contributing factors, including chronic infections with hepatitis B and C viruses, excess body weight, metabolic disorders such as diabetes, accumulation of fat in the liver (steatosis), tobacco use, and cirrhosis resulting from alcohol consumption [3]. Hepatocellular carcinoma (HCC) constitutes more than 90% of diagnosed cases, for which chemotherapy remains a predominant therapeutic strategy [4, 5]. Oral administration of anticancer agents such as sorafenib, a tyrosine kinase inhibitor, is commonly used for advanced-stage liver cancer [6–8]. However, the development of drug resistance during long-term chemotherapy remains a major cause of treatment failure and cancer recurrence [9–13].

**Fig. 1:**
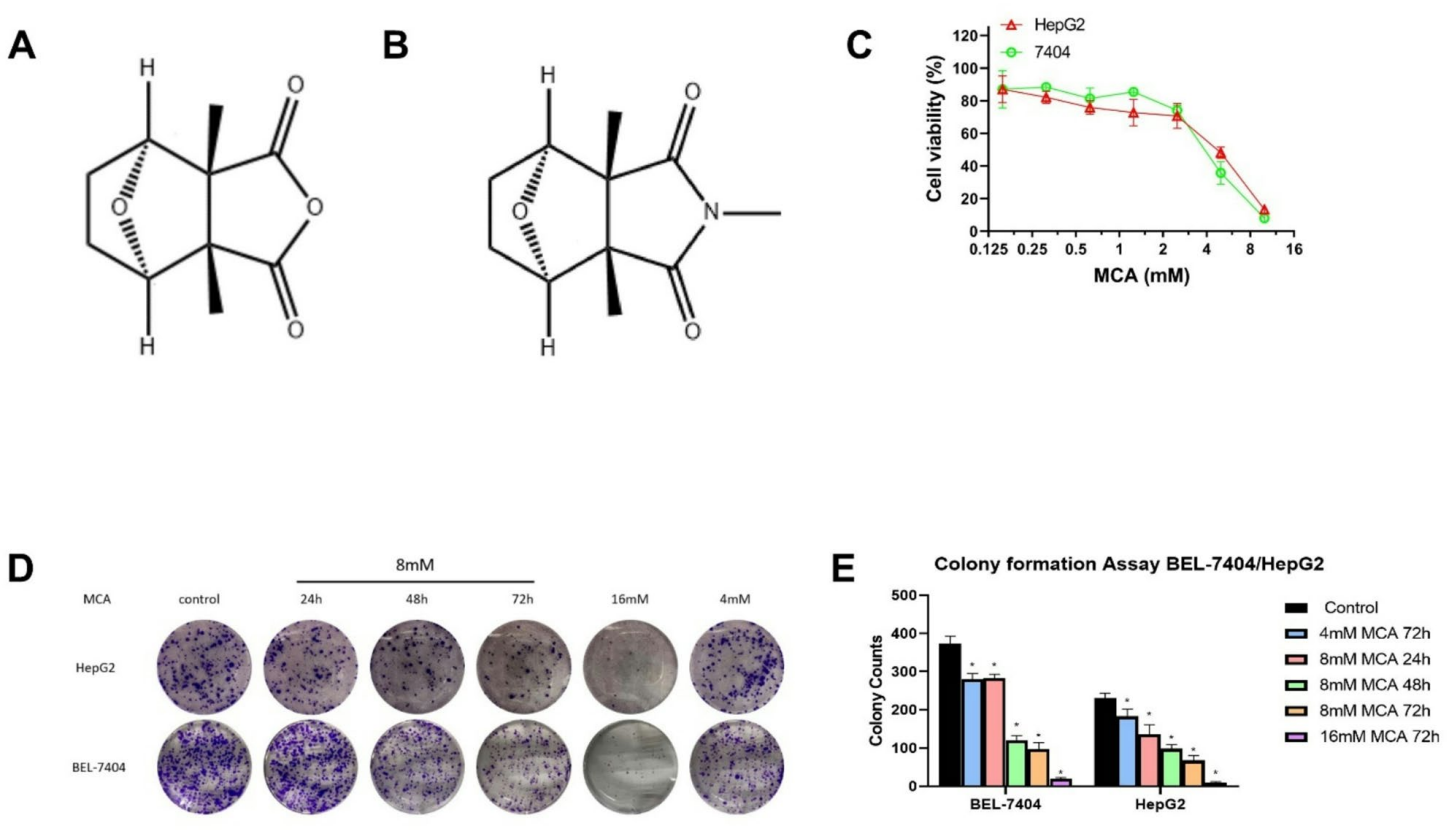
Chemical structures of (**A**) cantharidin and (**B**) methyl-cantharidimide. **C** Concentration-dependent cell viability curves showing the inhibitory effect of MCA on BEL-7404 and HepG2 cells, as assessed by the MTT assay. **D** and **E** Representative images and quantitative analysis of colony formation in BEL-7404 (**D**) and HepG2 (**E**) cells following treatment with increasing concentrations of MCA (4, 8, and 16 mM for 72 h)

Cantharidin (CTD) is a bioactive compound isolated from *Mylabris phalerata*, commonly known as the Chinese blister beetle [14], with its chemical structure shown in Fig. 1A. Traditionally used in Chinese medicine for over two millennia, CTD has been used to manage diverse medical conditions, including bacterial and fungal infections, molluscum contagiosum, skin warts, and several types of malignancies [15–20]. In recent years, CTD has shown potent anticancer activity against multiple tumor types, such as lung, bladder, oral, and renal cancers, as well as epidermoid carcinoma [21–27]. Although CTD exhibits promising therapeutic efficacy, its clinical application is restricted due to pronounced toxicity and significant side effects [27].

To address these challenges, various structurally modified derivatives of CTD have been developed to reduce its toxicity while retaining anticancer efficacy, thereby improving their suitability for clinical application [28]. One such analog, 2,3a,7a-trimethyl-hexahydro-4,7-epoxido-isoindol-1,3-dione—commonly known as methyl-cantharidimide (MCA)—has demonstrated lower toxicity than CTD while preserving its anticancer efficacy [29]. The molecular structure of MCA is illustrated in Fig. 1B. Prior studies have shown that MCA can trigger cell cycle arrest and promote programmed cell death in liver cancer cells, while exhibiting notably lower toxicity in non-cancerous hepatic cells [29]. Our previous study demonstrated that the anticancer efficacy of MCA is not compromised in ABCB1- and ABCG2-overexpressing, or cisplatin-resistant cancer cells [30]. However, the precise mechanisms underlying MCA’s anticancer activity remain to be fully elucidated.

This study primarily aims to investigate how MCA exerts its biological effects in liver cancer cells, with a focus on its influence on programmed cell death, cell cycle dynamics, generation of reactive oxygen species (ROS), and the molecular signaling mechanisms that may mediate these processes.

## Materials and methods

### Chemicals and reagents

MCA was generously supplied by Sihuan Bioengineering Co., Ltd. (Jiangsu, China). Cell culture reagents, including Dulbecco’s Modified Eagle Medium (DMEM), fetal bovine serum (FBS), bovine serum albumin (BSA), penicillin/streptomycin, and 0.25% trypsin, were obtained from Corning Incorporated (Corning, NY, USA). Dimethyl sulfoxide (DMSO) and the MTT reagent were purchased from Sigma-Aldrich (St. Louis, MO, USA), N-acetylcysteine (NAC) was purchased from Thermo Fisher Scientific (Waltham, MA, USA), and Dinaciclib was obtained from MedChemExpress LLC (Monmouth Junction, NJ, USA).

Primary antibodies targeting CDK1/Cdc25C (#4688), AIF (#5318), KEAP1 (#8047), PGAM5 (#24584), β-Actin (#4970), Bax (#2772), Bcl-2 (#4223), and HRP-linked anti-rabbit IgG (#7074) were sourced from Cell Signaling Technology Inc. (Danvers, MA, USA). The phospho-specific antibody against AIFM1 (Ser116) was provided by ECM Biosciences (Aurora, CO, USA). Reagents for functional assays included Click-iT™ฏ Plus EdU Alexa Fluor™ฏ 488 Flow Cytometry Assay Kit (Thermo Fisher Scientific, Waltham, MA, USA) with FxCycle™ฏ PI/RNase Staining Solution (Thermo Fisher Scientific), FITC Annexin V Apoptosis Detection Kit with PI (Biolegend Inc., San Diego, CA, USA), the Viability/Cytotoxicity Assay Kit (Biotium Inc., Fremont, CA, USA) and the DCFDA/H2DCFDA Cellular ROS Assay Kit (Abcam Inc., Waltham, MA, USA). Fluorescent reagents such as Alexa Fluor 549-labeled secondary antibodies and DAPI were acquired from Thermo Fisher Scientific Inc. (Waltham, MA, USA), which also supplied additional chemicals used in this study.

### Cell lines and cell culture

The human hepatocellular carcinoma cell line BEL-7404 was used to explore the antitumor mechanisms of MCA. Additionally, the HepG2 hepatoblastoma cell line was employed alongside BEL-7404 to support mechanistic investigations [31]. All cell lines were cultured in Dulbecco’s Modified Eagle Medium (DMEM) supplemented with 10% fetal bovine serum (FBS) and 1% penicillin-streptomycin. Cultures were maintained at 37 °C in a humidified incubator with 5% CO₂. Prior to treatment, cells were grown under drug-free conditions for a minimum of 14 days to ensure stable adaptation.

### Cytotoxicity assay

The MTT colorimetric assay was utilized to assess both cell viability and resistance, following a previously described approach [32]. Cells were initially trypsinized using 0.25% trypsin-EDTA, suspended in culture medium, and distributed into 96-well plates at a density of 5 × 10³ cells per well in 180 µL of DMEM containing 10% FBS. After allowing cells to adhere overnight, various concentrations of MCA or comparator drugs were added, adjusting the total volume in each well to 200 µL. After a 72-hour treatment period, MTT reagent (4 mg/mL) was introduced and incubated for an additional 4 h to enable formazan production. Supernatants were aspirated, and 100 µL of DMSO was added to dissolve the resulting crystals under mild shaking for 10 min. Absorbance readings were captured at 570 nm using a Dynex Technologies microplate spectrophotometer (Chantilly, VA). The IC₅₀ for each compound was calculated by generating viability curves and analyzed using the Bliss independence model [33].

### Colony formation assay

A colony formation assay was carried out to determine the suppressive effects of MCA on the proliferation of liver cancer cells. BEL-7404 and HepG2 cells were plated into 6-well plates at a density of 500 cells per well in 2 mL of complete medium. After allowing the cells to adhere overnight, they were exposed to MCA in either a dose-dependent fashion (4, 8, and 16 mM for 72 h) or a time-course format using a constant dose (8 mM for 24, 48, or 72 h). Upon completion of treatment, the drug-containing media were replaced with fresh DMEM, and cells were incubated for an additional 10 days, with the medium refreshed every two days. Colonies were then fixed using 4% paraformaldehyde and stained with 0.5% crystal violet solution. Images were captured with a Nikon Eclipse Ts2R-FL microscope (Melville, NY), and colony counts were obtained based on the captured fields.

### Western blotting

Western blotting was used to assess treatment-induced changes in protein expression. Cells were lysed on ice for 20 min using RIPA buffer (Thermo Fisher Scientific Inc., Rockford, IL, USA), followed by centrifugation at 12,000 × g for 20 min at 4 °C to remove debris. The supernatant containing total proteins was collected, and protein concentration was determined using the Pierce BCA assay kit (Thermo Fisher Scientific Inc., Rockford, IL, USA). Equal protein samples were separated by SDS-polyacrylamide gel electrophoresis (SDS-PAGE) and transferred to PVDF membranes. To block nonspecific binding, membranes were incubated in 5% non-fat milk diluted in TBST for 2 h at room temperature. Primary antibodies were applied and incubated overnight at 4 °C. After washing, membranes were treated with HRP-conjugated secondary antibodies for 2 h at room temperature. Signals were visualized with enhanced chemiluminescence (ECL), and band intensities were quantified using ImageJ.

### Immunofluorescence assay

BEL-7404 cells were seeded into 24-well plates at a density of 1 × 10⁴ cells per well and incubated overnight to allow for attachment. The following day, cells were treated with varying concentrations of MCA (2, 4, or 8 mM) for 72 h. After completion of drug exposure, cells were fixed using 4% paraformaldehyde and subsequently permeabilized with 0.25% Triton X-100. To block nonspecific antibody binding, a 6% bovine serum albumin (BSA) solution was applied for 1 h. Primary monoclonal antibody against CDK1 was then added and incubated overnight at 4 °C. After washing steps, cells were treated with a secondary antibody conjugated to Alexa Fluor 549 in the dark for 2 h. Nuclei were stained with DAPI, and fluorescence imaging was performed using a Nikon Eclipse Ts2R-FL microscope (Melville, NY, USA). The fluorescence intensity was quantified using ImageJ.

### Reverse transcription-quantitative PCR (RT-qPCR)

Total RNA was extracted using a commercial RNA isolation kit (Thermo Fisher Scientific Inc., Rockford, IL, USA) in accordance with the supplier’s protocol. RNA concentration and purity were evaluated by spectrophotometric measurement at 260 nm, and the OD260/OD280 ratio was calculated to assess sample quality. First-strand cDNA synthesis was performed using the SuperScript™ II Reverse Transcriptase Kit (Invitrogen™). Quantitative real-time PCR was then conducted using SYBR Select Master Mix (Applied Biosystems, Foster City, CA, USA) with gene-specific primers listed in Table S1. Amplification reactions were run on an AriaMx Real-Time PCR System (Agilent Technologies, Santa Clara, CA, USA). Relative mRNA levels were determined by the 2^−ΔΔCt method, using GAPDH as the internal control for normalization.

### Cell cycle analysis

BEL-7404 and HepG2 cells were treated with 8 mM MCA for 72 h. Following exposure, cells were collected, treated with the Click-iT™ฏ Plus EdU Alexa Fluor™ฏ 488 Flow Cytometry Assay Kit (Thermo Fisher Scientific, Waltham, MA, USA). Cell cycle distribution was analyzed on an Invitrogen™ Attune™ NxT Flow Cytometer (Thermo Fisher Scientific, Waltham, MA, USA), with 10,000 events acquired per sample at a low flow rate. The proportions of cells in each phase of the cell cycle were quantified using the FlowJo (Becton Dickinson & Company) analysis software.

### Intracellular ROS assay

BEL-7404 and HepG2 cells were seeded in 96-well culture plates at a density of 3 × 10⁴ cells per well. After 24 h of incubation in complete growth medium, intracellular ROS levels were assessed using the DCFDA Cellular ROS Assay Kit (Abcam), following the manufacturer’s protocol. Post-staining, cells were treated with MCA at final concentrations of 2, 4, or 8 mM for 4 h. Tert-butyl hydroperoxide (TBHP) served as the positive control. After the treatment period, fluorescent images were obtained using a Nikon Eclipse Ts2R-FL fluorescence microscope (Melville, NY, USA). The fluorescence intensity was quantified using ImageJ.

### Cell apoptosis analysis

BEL-7404 and HepG2 cells were exposed to MCA at concentrations of 8mM alone or combined treatment with 1mM NAC for 72 h. After treatment, cells were collected and processed using the FITC Annexin V Apoptosis Detection Kit (Biolegend Inc., San Diego, CA, USA) according to the manufacturer’s protocol. Apoptotic populations were quantified on an Invitrogen™ Attune™ NxT Flow Cytometer (Thermo Fisher Scientific, Waltham, MA, USA), acquiring 10,000 events per sample. The percentage of live, apoptotic, and necrotic cells was quantified using FlowJo (Becton Dickinson & Company) analysis software.

### Reverse phase protein array (RPPA)

BEL-7404 cells were exposed to either vehicle or 8 mM MCA for 72 h. Post-treatment, total proteins were extracted, quantified, and diluted in serial steps before submission for reverse-phase protein array (RPPA) analysis. This procedure was carried out by the Mills Institute for Personalized Cancer Care at Fynn Biotechnologies Ltd. (Shandong, China) [34]. The RPPA workflow included spotting the diluted protein lysates onto nitrocellulose-coated chips, followed by sequential incubation with primary and secondary antibodies. Luminescence signals were scanned and quantified, and expression levels were normalized accordingly. The final output provided a comparative protein expression profile of cancer-associated targets between control and MCA-treated groups.

### 3D multicellular tumor spheroids model

BEL-7404 and HepG2 cells were seeded at 1,500 cells per well in Nunclon Sphera-treated, U-bottom 96-well microplates. Plates were centrifuged at 500 rpm for 10 min and incubated overnight at 37 °C to allow spheroid formation. The resulting 3D spheroids were then treated with MCA at concentrations of 2, 4, and 8 mM for 10 days. Images of spheroids were captured every three days following drug addition using a Nikon Eclipse Ts2R-FL microscope (Melville, NY). On the final day of treatment, spheroid viability was assessed using a live/dead cell viability/cytotoxicity assay kit (Biotium Inc., Fremont, CA). Stained spheroids were imaged using the same microscope, and live/dead cells were visualized via merged fluorescence channels. The fluorescence intensity was quantified using ImageJ.

### Tumor xenograft mouse model

To establish xenograft tumors, HepG2 cells (2 × 10⁶) were suspended in 200 µL of Cultrex Stem Cell Qualified RGF Basement Membrane Extract (R&D Systems™, Minnesota, USA) and subcutaneously injected into male athymic NCR nude mice. The animals were then randomly assigned to one of four groups: high-dose MCA (100 mg/kg, intraperitoneal), low-dose MCA (50 mg/kg, i.p.), a positive control group receiving Dinaciclib (40 mg/kg, i.p.), and a vehicle control group. Injections were administered every three days for a total of six doses. Tumor size and body weight were monitored at 3-day intervals using digital calipers. At the study endpoint, mice were euthanized via CO₂ inhalation, and tumors were excised and weighed. Tumor volume was calculated based on a previously established formula [35].

### Statistical analysis

All statistical evaluations were conducted using GraphPad Prism version 8 (GraphPad Software, La Jolla, CA, USA). Results are presented as mean values accompanied by standard deviation (SD). To assess differences among multiple experimental groups, one-way analysis of variance (ANOVA) was applied, followed by Tukey’s post hoc test for pairwise comparisons. A p-value below 0.05 was regarded as statistically significant.

## Result

### MCA inhibits colony formation in liver cancer BEL-7404 and HepG2 cells

As illustrated in Fig. 1A, treatment with MCA led to a comparable decline in cell viability in both BEL-7404 and HepG2 liver cancer cell lines, as determined by the MTT assay. IC₅₀ of MCA was determined to be 7.83 ± 0.86 mM in BEL-7404 cells and 8.66 ± 0.93 mM in HepG2 cells. To further explore MCA’s antiproliferative potential, a colony formation assay was conducted using the same cell lines. Cells were exposed to MCA at increasing concentrations (4, 8, and 16 mM) for 72 h, as well as for varying durations (8 mM for 24, 48, and 72 h). As depicted in Fig. 1D, E and a notable decrease in both the number and size of colonies was observed in a dose- and time-dependent fashion in both cell models. These results indicate that MCA exerts a pronounced inhibitory effect on colony formation, which is correlated with the concentration of the compound and the length of exposure.

### Differential protein expression in liver cancer cells following MCA treatment

**Fig. 2:**
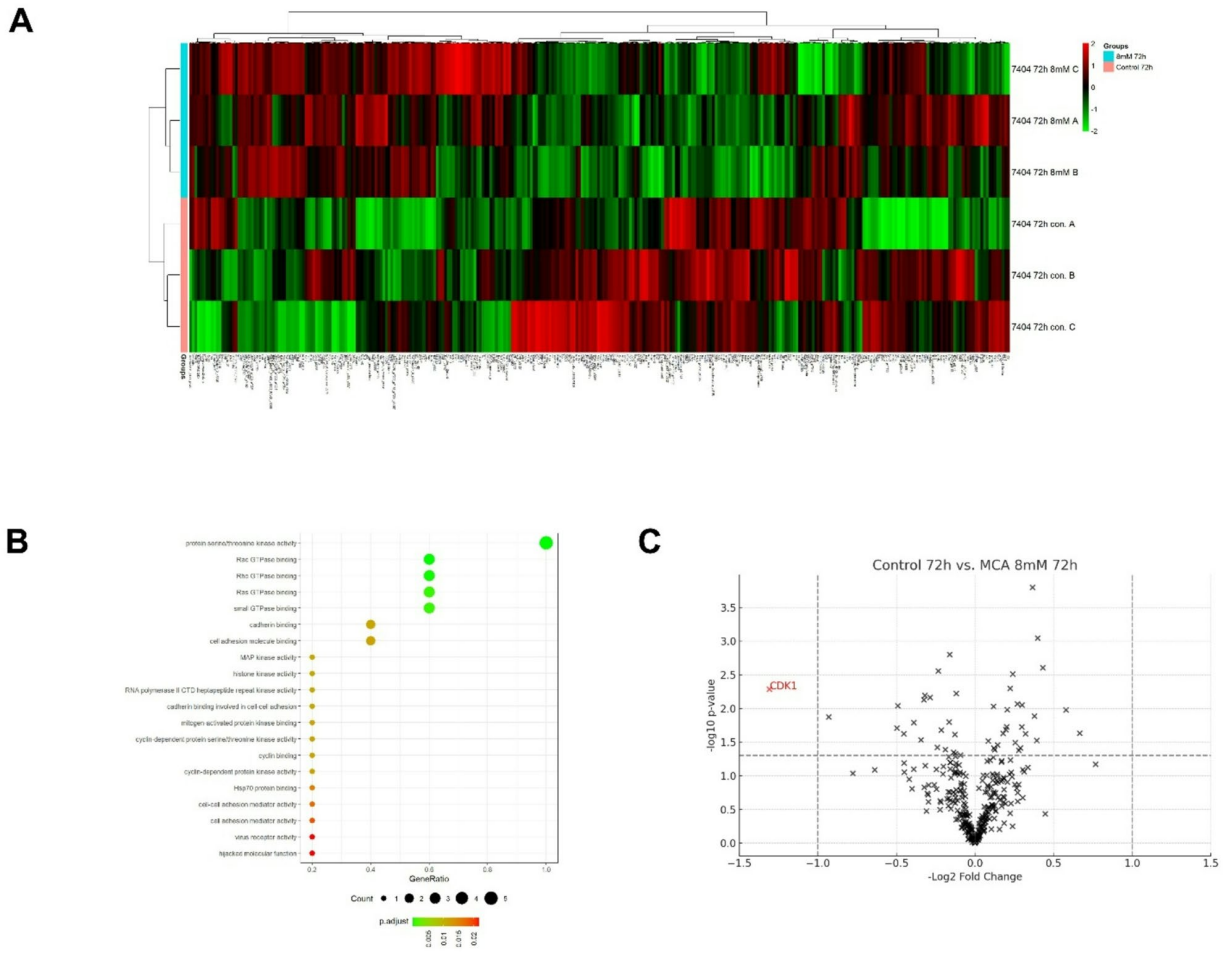
Bioinformatic analysis of protein expression in MCA-treated BEL-7404 cells using RPPA. **A** Heatmap showing the differentially expressed protein profiles between MCA-treated (8 mM, 72 h) and vehicle control groups in BEL-7404 liver cancer cells. **B** Gene Ontology (GO) dot plot illustrating the top enriched GO terms based on differentially expressed proteins between the MCA-treated and control groups. **C** Volcano plot depicting significantly upregulated and downregulated proteins in the MCA-treated group compared to the control. Proteins with adjusted *p* < 0.05 and fold change > 2 or < 0.5 were considered significantly differentially expressed

To investigate the molecular effects of MCA, reverse-phase protein array (RPPA) profiling was conducted in BEL-7404 liver cancer cells following treatment with 8 mM MCA for 72 h. The analysis identified changes in protein expression by comparing MCA-treated cells with vehicle controls. A heatmap visualization was generated to highlight differentially expressed proteins between the two groups. As shown in Fig. 2A, most differentially expressed proteins exhibited opposing regulatory patterns between the two groups. Additionally, a Gene Ontology (GO) dot plot was generated to illustrate the top 20 enriched GO terms between the treatment and control groups (Fig. 2B). Among these, serine/threonine kinase activity showed the most significant difference. These distinct protein expression profiles may contribute to the anticancer effects of MCA.

To further identify the most significantly altered proteins (padj < 0.05 with fold change > 2 or < 0.5), a volcano plot was generated to visualize the differential protein expression between MCA-treated and control cells. As shown in Fig. 2C, cyclin-dependent kinase 1 (CDK1) was significantly downregulated (more than 2-fold) in MCA-treated BEL-7404 cells compared to the control. CDK1, a serine/threonine protein kinase, plays a pivotal role in regulating the cell cycle by partnering with Cyclin B1 to drive the transition from the G2 phase to mitosis [36, 37]. This finding suggests that downregulation of CDK1 may be a critical component of MCA’s mechanism of anticancer activity.

### MCA induced CDK1 downregulation in liver cancer BEL-7404 and HepG2 cells

**Fig. 3:**
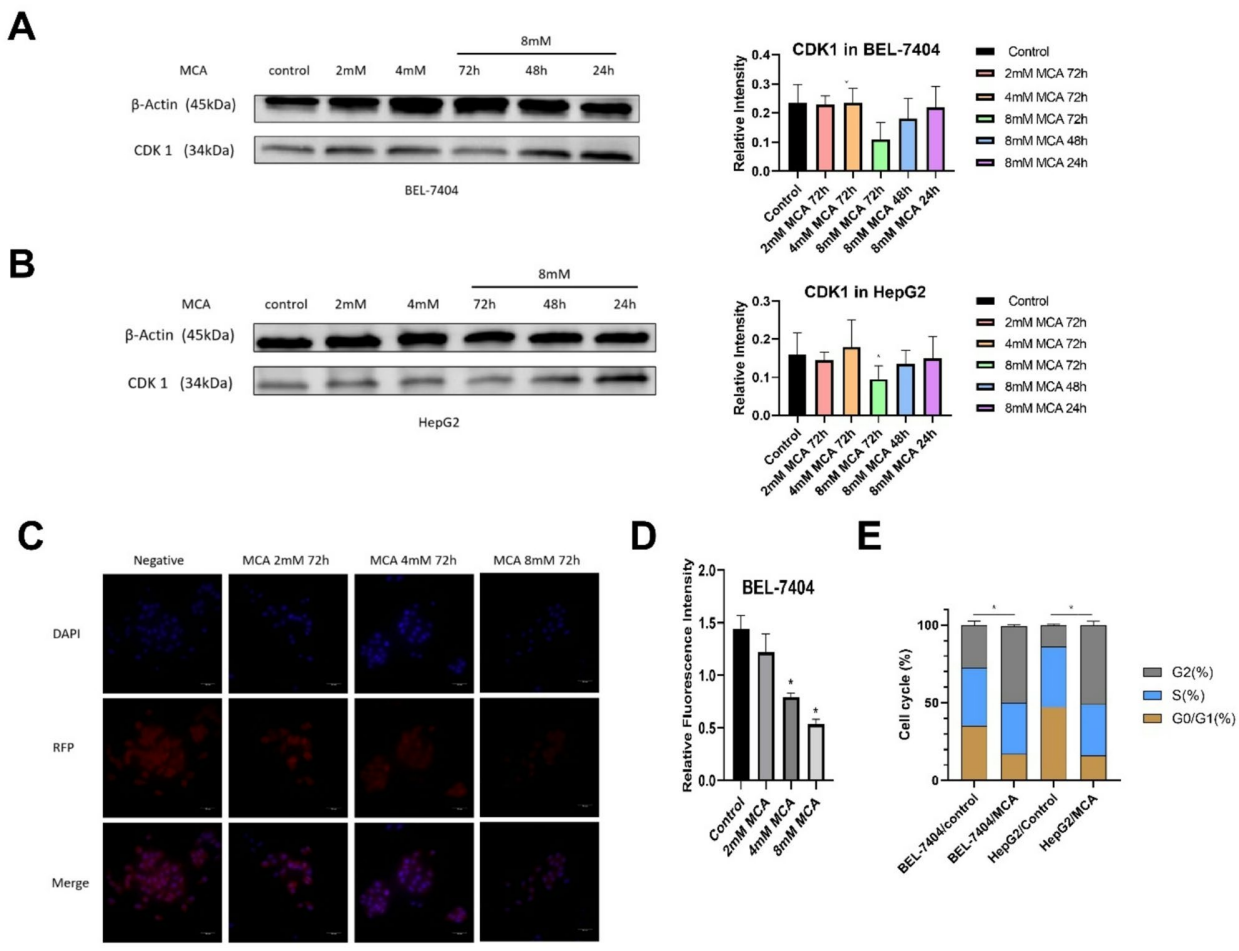
Effect of MCA on CDK1 expression, cell cycle and subcellular localization in liver cancer cells. **A**, **B** Western blot analysis of CDK1 protein expression in BEL-7404 (**A**) and HepG2 (**B**) cells treated with increasing concentrations of MCA (2, 4, and 8 mM) for 72 h. **C** Immunofluorescence analysis of CDK1 subcellular localization in BEL-7404 cells after 72-hour treatment with MCA (2, 4, and 8 mM). CDK1 was detected using Alexa Fluor 549-conjugated secondary antibody (red), and nuclei were counterstained with DAPI (blue). **D** Quantification of relative fluorescence intensity was performed using ImageJ software and expressed as the ratio of CDK1 signal to DAPI nuclear staining. **E** Cell cycle distribution analysis showing the effects of 72-hour treatment with 8 mM MCA on BEL-7404 and HepG2 cells

Based on the RPPA results, regulation of CDK1 expression may be one of the key mechanisms underlying MCA’s anticancer activity. To confirm the alteration of CDK1 expression, Western blot analysis was performed in BEL-7404 and HepG2 liver cancer cells. Both cell lines were treated with MCA at concentrations of 2, 4, and 8 mM for 72 h. A time-dependent study was also conducted by treating cells with 8 mM MCA for 24, 48, or 72 h. As shown in Fig. 3A and B, CDK1 expression was significantly downregulated after 72 h of treatment with 8 mM MCA in both BEL-7404 and HepG2 cells. Nonetheless, the treatment groups exposed to reduced doses or shorter incubation periods did not exhibit any statistically meaningful alterations.

**Fig. 4:**
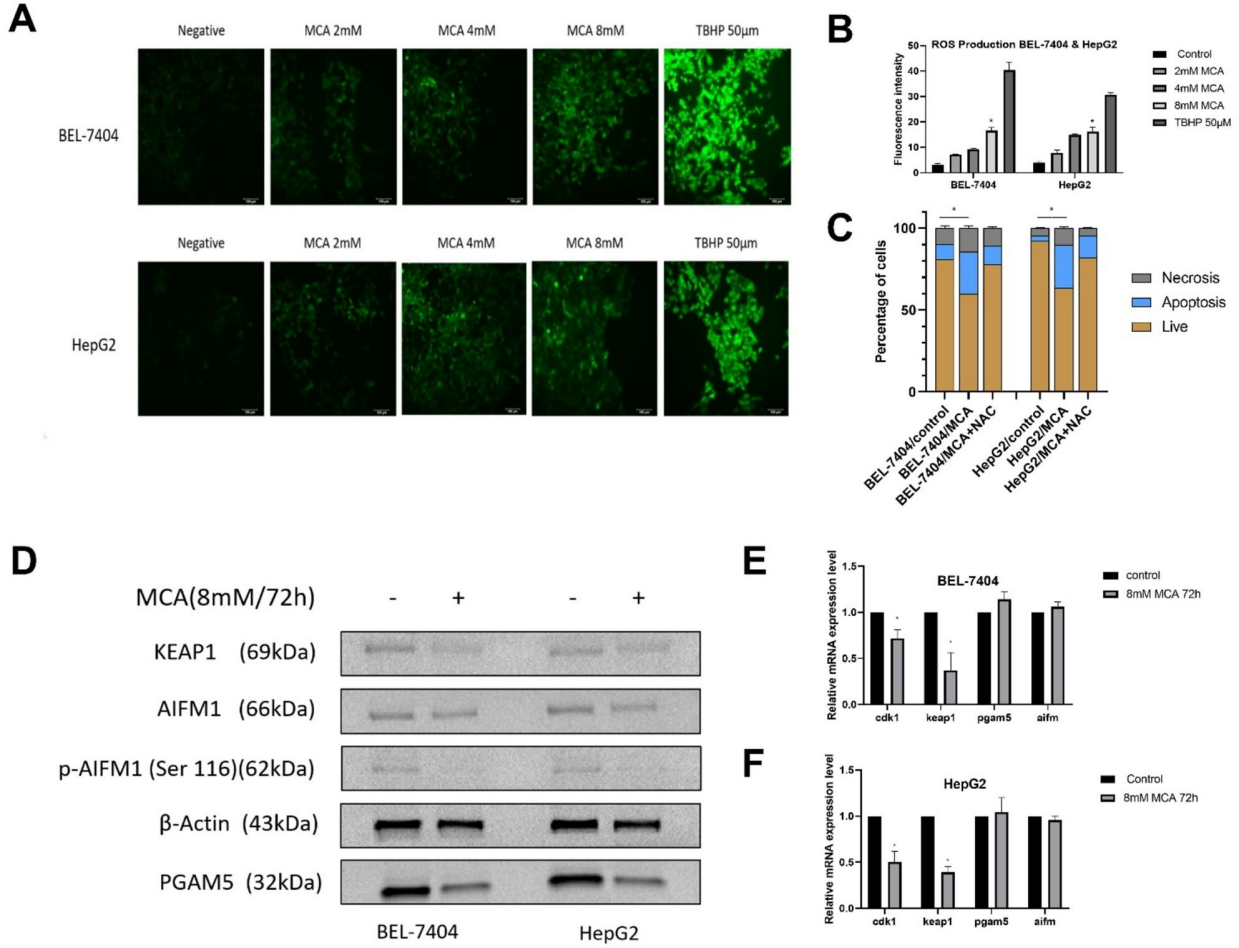
Effect of MCA on ROS production and oxeiptosis-related pathways in liver cancer cells. **A** MCA-induced ROS production in BEL-7404 and HepG2 cells after 4-hour treatment at varying concentrations (2, 4, and 8 mM), assessed using the DCFDA fluorescence assay. Tert-Butyl Hydroperoxide (TBHP) was used as positive control. Quantitative analysis of DCFDA fluorescence intensity corresponding to ROS levels shown in panel (**B**). **C** Evaluation of ROS-dependent apoptosis using the antioxidant N-acetylcysteine (NAC) in BEL-7404 and HepG2 cells. Cells were treated with vehicle control, 8 mM MCA alone, or 8 mM MCA combined with 1 mM NAC for 72 h. **D** Western blot analysis of oxeiptosis-related protein markers (KEAP1, PGAM5, AIFM1, p-AIFM1) in BEL-7404 and HepG2 cells following 72-hour treatment with 8 mM MCA. **E**, **F** Relative mRNA expression levels of KEAP1, PGAM5, and AIFM1 in BEL-7404 (**E**) and HepG2 (**F**) cells after 72-hour treatment with 8 mM MCA, measured by qRT-PCR

To further investigate whether MCA also affects CDK1 expression at the transcriptional level, qRT-PCR was performed on BEL-7404 and HepG2 cells treated with 8 mM MCA for 72 h, along with a vehicle-treated control group. Figure 4E and F demonstrate a notable decline in CDK1 mRNA levels in both liver cancer cell lines after MCA exposure. This suggests that MCA effectively suppresses CDK1 expression at both the transcriptional and protein levels in these cells.

### Effect of MCA on the localization of CDK1 in liver cancer cells

As CDK1 is involved in cell cycle regulation, its activation is typically associated with nuclear accumulation [38]. To investigate whether MCA influences the intracellular distribution of CDK1 in liver cancer cells, an immunofluorescence staining assay was conducted in BEL-7404 cells after a 72-hour exposure to MCA at concentrations of 2, 4, and 8 mM. As illustrated in Fig. 3C, and 3D, treatment with 4 mM and 8 mM MCA led to a noticeable reduction in red fluorescence intensity, indicating decreased CDK1 expression compared to untreated controls. Despite this decline in signal, the spatial distribution pattern of CDK1 remained largely unchanged across all treatment conditions; the protein continued to exhibit a cytoplasmic localization consistent with that observed in the control group.

### MCA arrested BEL-7404 and HepG2 liver cancer cells in the G2/M phase

To determine whether MCA-mediated suppression of CDK1 influences cell-cycle regulation in liver cancer cells, we examined cell-cycle profiles in BEL-7404 and HepG2 cells following treatment. Exposure to 8 mM MCA resulted in a pronounced enrichment of cells at the G2/M checkpoint, suggesting an interruption of normal progression into the G1 phase (Fig. 3E and Table S2). In both cell lines, the fraction of cells accumulating in the G2/M phase was significantly increased relative to vehicle-treated controls (*p* < 0.05).

### MCA is not involved in the Bcl-2 and Bax apoptosis pathway

To determine the mechanisms of MCA’s apoptosis-inducing efficacy shown in the previous study [30], Western blotting analysis was performed in BEL-7404 cells to observe Bcl-2 and Bax expression levels. Bcl-2 and Bax are a pair of typical upstream regulators of caspase apoptosis pathway [39]. Apoptosis is often triggered by an imbalance between pro-apoptotic and anti-apoptotic proteins, notably the upregulation of Bax and downregulation of Bcl-2 [39, 40]. To examine whether MCA influences this pathway, BEL-7404 cells were exposed to MCA at concentrations of 2, 4, and 8 mM for 72 h. Additionally, a time-course study was conducted using 8 mM MCA for 24, 48, and 72 h. As presented in Supplementary Figure 1, MCA treatment did not result in substantial changes in Bcl-2 or Bax protein expression. These findings suggest that MCA-induced apoptosis in BEL-7404 cells is unlikely to proceed via the classical Bcl-2/Bax-dependent caspase pathway.

### MCA induces ROS production in liver cancer cells

To explore the mechanism by which MCA may induce apoptosis, intracellular ROS levels were measured in BEL-7404 and HepG2 cells using DCFDA staining. After a 4-hour incubation with MCA at concentrations of 2, 4, and 8 mM, cells were processed using the ROS-sensitive fluorescent probe DCFDA. This compound becomes fluorescent upon oxidation by intracellular ROS, forming 2′,7′-dichlorofluorescein (DCF). The resulting fluorescence intensity reflects the extent of ROS accumulation following MCA exposure. As shown in Fig. 4A and B, fluorescence intensity increased with higher MCA concentrations in both BEL-7404 and HepG2 cells. These results indicate that MCA induces ROS production in a concentration-dependent manner. To verify whether MCA-triggered apoptosis is mediated by reactive oxygen species (ROS), a rescue experiment was conducted using the antioxidant NAC in combination with apoptosis analysis [41]. As illustrated in Fig. 4C, simultaneous treatment with 8 mM MCA and 1 mM NAC for 72 h significantly reduced the proportion of apoptotic cells in both BEL-7404 and HepG2 cell lines compared with cells treated with 8 mM MCA alone. Consequently, MCA-induced apoptosis may be associated, at least in part, by ROS generation.

### MCA induces oxeiptosis in liver cancer cells

Since our results indicated that MCA does not induce apoptosis through the Bcl-2/Bax-regulated, caspase-dependent pathway (supplementary Figure 1), and given that MCA treatment induces ROS production, we hypothesized that MCA may promote apoptosis via an oxidative stress-related mechanism. Oxeiptosis is a form of regulated cell death that operates independently of caspases and is initiated by elevated levels of ROS [42]. To evaluate the involvement of this pathway, we analyzed the expression and phosphorylation status of key oxeiptosis-related proteins—KEAP1, phosphorylated AIFM1 at Ser116 (p-AIFM1), and PGAM5—in BEL-7404 and HepG2 cells treated with 8 mM MCA for 72 h. These results were compared to those from untreated control cells.

The NRF2–PGAM5–AIFM1 axis plays a critical role in the activation of oxeiptosis. Elevated oxidative stress can lead to the degradation of KEAP1, the stabilizer of NRF2, resulting in the release and mitochondrial translocation of PGAM5 [43]. PGAM5 then dephosphorylates p-AIFM1 (Ser116), leading to the release of active AIFM1, which initiates downstream cell death pathways [44]. Western blot analysis showed that MCA treatment significantly downregulated KEAP1 expression in both BEL-7404 and HepG2 cells and led to dephosphorylation of p-AIFM1 (Ser116) (Fig. 4D). However, no notable differences in total protein levels of AIFM1 and PGAM5 were observed between MCA-treated cells and the untreated control group.

To further assess the involvement of oxeiptosis at the transcriptional level, qRT-PCR was performed on BEL-7404 and HepG2 cells treated with 8 mM MCA for 72 h. As shown in Fig. 4E and F, following MCA treatment, a marked decrease in KEAP1 mRNA expression was observed in both BEL-7404 and HepG2 cells. Conversely, transcript levels of PGAM5 and AIFM1 showed no significant difference relative to the control. These findings imply that MCA may promote cell death in liver cancer cells, at least partially, by triggering the oxeiptosis pathway.

### Evaluation of MCA toxicity in liver cancer multicellular tumor spheroids (MCTSs)

To further investigate the antitumor potential of MCA in a more physiologically relevant model, a multicellular tumor spheroid (MCTS) system was utilized. This 3D culture system more accurately reflects the structural and physiological complexity found in solid tumors. Unlike traditional two-dimensional (2D) monolayer cultures, MCTSs display altered responsiveness to chemotherapeutic agents, largely due to restricted drug diffusion and penetration within the compact spheroid structure [45]. BEL-7404 and HepG2 spheroids were generated and treated with MCA at concentrations of 2, 4, and 8 mM for up to 10 days. At the end of the treatment phase, cell viability within the spheroids was evaluated using a live/dead staining method. Calcein AM, which fluoresces green, marked viable cells, whereas EthD-1, emitting red fluorescence, identified non-viable cells—allowing visualization of MCA-induced cytotoxicity.

**Fig. 5:**
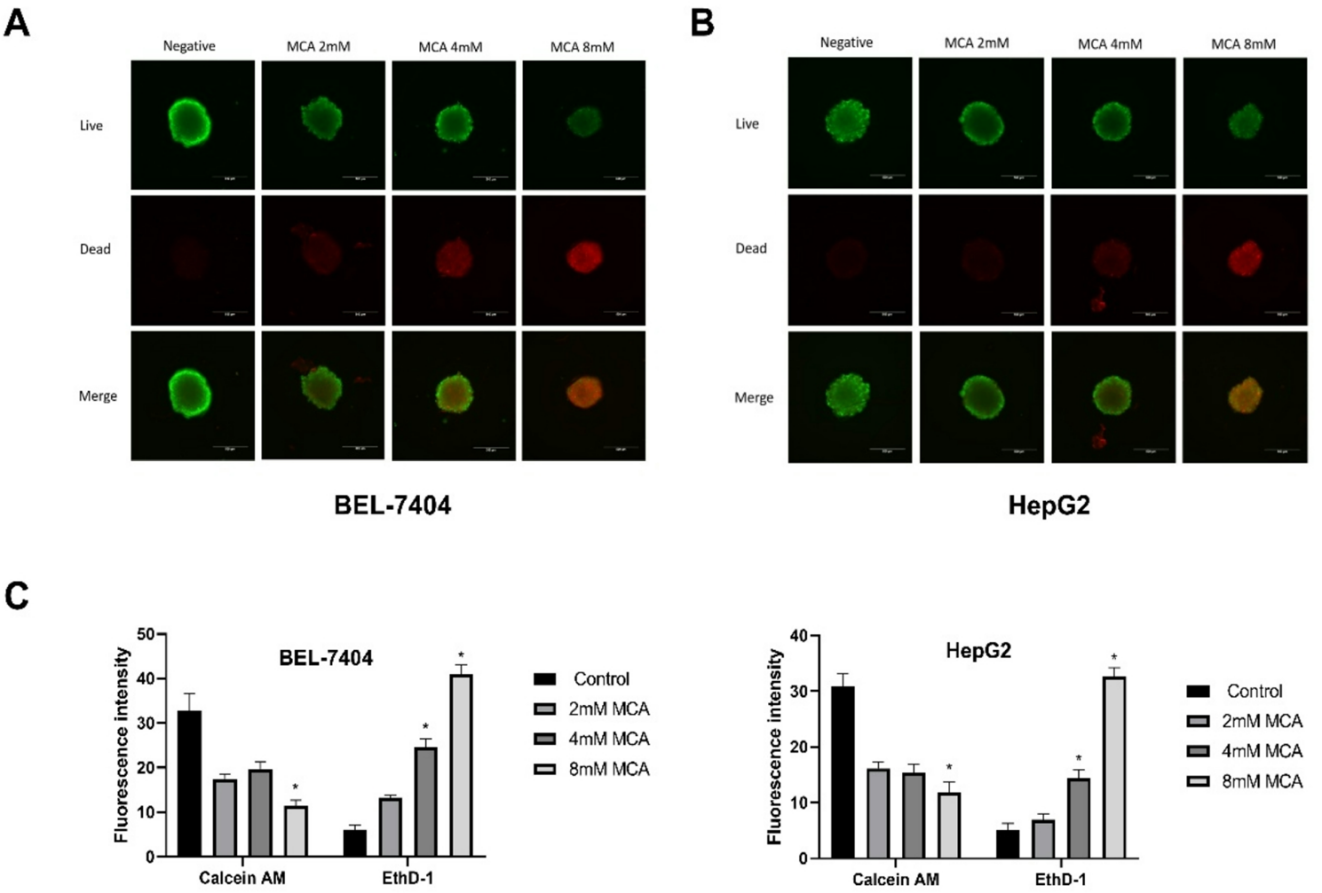
MCA-induced growth inhibition and cell death in multicellular tumor spheroids (MCTSs). Representative images of MCTSs formed by BEL-7404 (**A**) and HepG2 (**B**) cells after 10 days of treatment with MCA (2, 4, and 8 mM). Live/dead cell staining was performed using Calcein AM (green, live cells) and EthD-1 (red, dead cells). MCA treatment led to a dose-dependent increase in cell death and reduction in spheroid size. **C** Quantitative comparison of Calcein AM and EthD-1 fluorescence intensity across the different treatment groups

After 10 days of MCA treatment, BEL-7404 and HepG2 spheroids showed a higher proportion of dead cells and fewer live cells in the 4 mM and 8 mM treatment groups compared to the control group (Fig. 5A, B and C). Additionally, the diameter of spheroids in the 8 mM treatment group was significantly reduced relative to the control, indicating that MCA treatment impaired spheroid growth. These results demonstrate that MCA retains its anticancer efficacy—inducing both cell death and growth inhibition—in the MCTS models of BEL-7404 and HepG2 cells.

### Evaluation of the MCA’s anticancer efficacy in the tumor xenograft mice model using HepG2 cell line

**Fig. 6:**
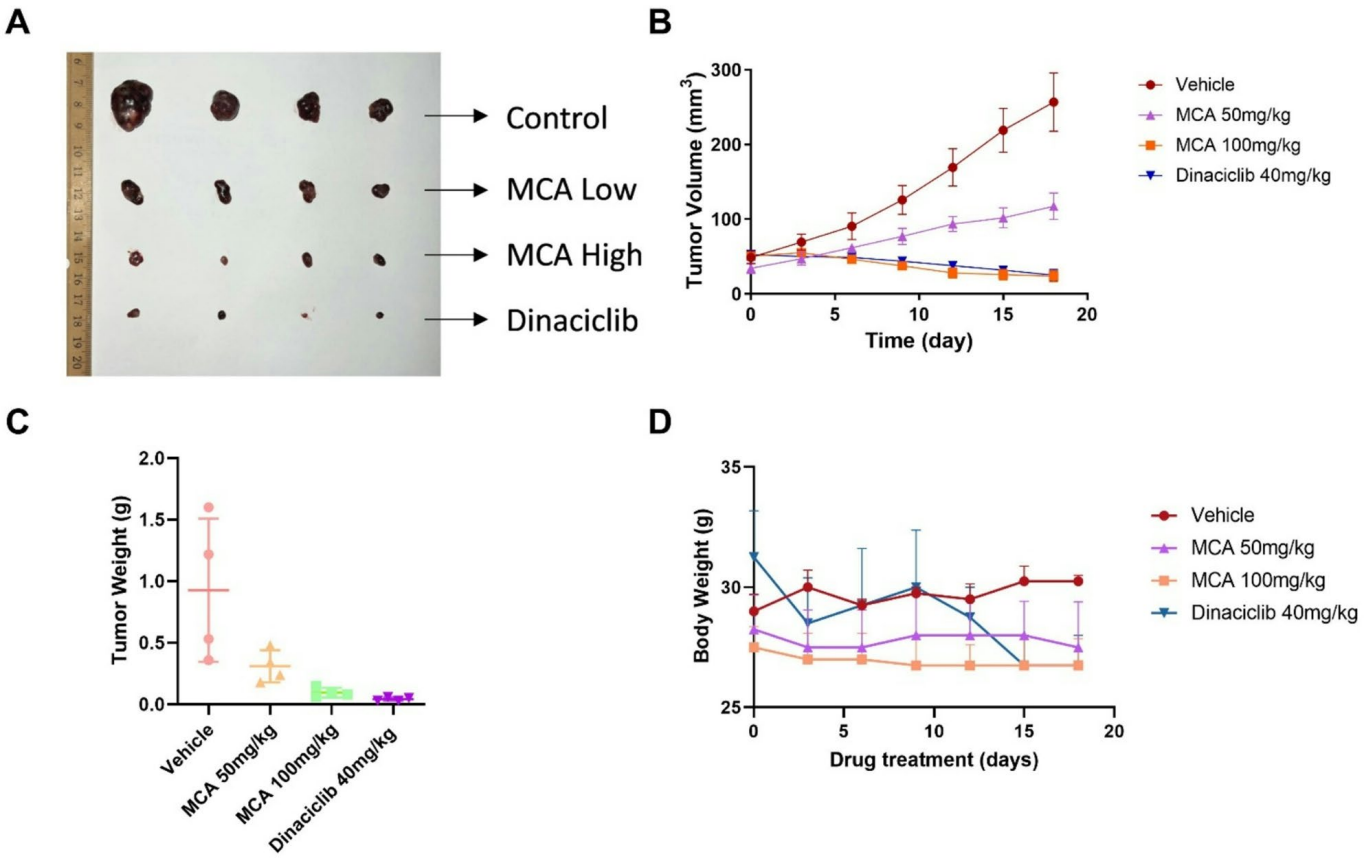
Anticancer efficacy of MCA in a HepG2 xenograft mouse model. **A** Representative images of resected tumors from each group on day 18. Tumor volume (**B**) and tumor weight (**C**) at the end of the treatment period. **D** Body weights of mice in each group were recorded every three days throughout the study to monitor systemic toxicity

To assess the in vivo anticancer potential of MCA, a xenograft model was developed using HepG2 liver cancer cells in nude mice. Animals were randomly assigned to one of four treatment arms: MCA at a high dose (100 mg/kg), MCA at a low dose (50 mg/kg), the CDK1 inhibitor Dinaciclib (40 mg/kg) as a positive control, or a vehicle control. Dinaciclib served as a reference compound due to its known inhibitory effect on CDK1 [46]. Figure 6A displays representative images of tumors collected from each treatment group.

Following 18 days of administration, all treatment groups—MCA at 100 mg/kg, MCA at 50 mg/kg, and Dinaciclib at 40 mg/kg—exhibited markedly decreased tumor volumes and weights relative to the vehicle-treated controls (Fig. 6B and C). As anticipated, the group receiving 50 mg/kg of MCA demonstrated reduced efficacy compared to the higher MCA dose and the Dinaciclib group, likely due to the lower drug concentration. Importantly, no significant differences were observed in tumor volume and weight between the MCA 100 mg/kg group and the Dinaciclib-treated group, suggesting that MCA at a dose of 100 mg/kg offers comparable anticancer effects to Dinaciclib in this in vivo model.

As shown in Fig. 6D, body weights of mice in the vehicle, low-dose MCA, and high-dose MCA groups remained stable throughout the study, suggesting minimal adverse effects associated with MCA treatment. In contrast, mice treated with Dinaciclib experienced significant weight loss by day 18, indicating a higher level of toxicity.

In summary, MCA exhibited potent antitumor activity with minimal toxicity in the HepG2 xenograft mouse model, highlighting its promise as a potential therapeutic agent for liver cancer.

## Discussion

In this study, MCA inhibits colony formation in BEL-7404 and HepG2 liver cancer cells. The inhibition effect of MCA is stronger with higher concentration (16 mM compared with 8 mM and 4 mM for 72 h, respectively) and longer incubation time (8 mM for 72 h compared with 48 h and 24 h, respectively). The results suggest that MCA suppresses the growth of BEL-7404 and HepG2 liver cancer cells.

RPPA results demonstrated that CDK1 expression was downregulated in BEL-7404 liver cancer cells after treatment with 8 mM MCA for 72 h, compared to the vehicle control. CDK1 plays a pivotal role in cell cycle regulation by forming a complex with Cyclin B1, which facilitates the transition of cells into mitosis [37]. The activity of the Cyclin B1–CDK1 complex is modulated by phosphorylation at tyrosine 15 and threonine 14 residues on CDK1, which is catalyzed by the kinases WEE1 and MYT1 [47, 48]. As a result, decreased CDK1 expression can disrupt Cyclin B1–CDK1 complex formation, thereby inducing arrest at the G2/M phase of the cell cycle. This observation is consistent with prior studies reporting MCA-induced cell cycle blockade in liver cancer models [29].

Western blot analysis further validated that CDK1 protein levels were markedly diminished in both BEL-7404 and HepG2 cells after 72 h of exposure to 8 mM MCA. Consistently, quantitative real-time PCR (qRT-PCR) revealed a parallel decrease in CDK1 mRNA expression across both cell types. These findings collectively indicate that MCA suppresses CDK1 expression at both the gene transcription and protein synthesis levels, thereby contributing to the induction of cell cycle arrest in liver cancer cells.

Since the cyclin B1-CDK1 complex is intended to accumulate in the cell nucleus during the normal cell cycle [49], immunofluorescence assays were conducted in BEL-7404 cells to determine if the subcellular localization of CDK1 was modified by MCA. Following 72 h of treatment with MCA at concentrations of 2, 4, and 8 mM, CDK1 remained localized in the cytoplasm across all groups, showing no observable shift in subcellular distribution compared to the untreated control. However, a dose-dependent decrease in red fluorescence intensity was observed, particularly in the 8 mM MCA group, suggesting reduced CDK1 expression. These findings demonstrate that while MCA suppresses CDK1 levels in BEL-7404 cells, it does not impact the protein’s cytoplasmic localization. Additional cell-cycle profiling revealed that MCA exposure led to pronounced arrest at the G2/M checkpoint in liver cancer cells, an effect that is likely associated with suppression of CDK1 expression.

To investigate which apoptotic pathway may be influenced by MCA treatment, Western blot analysis was employed to assess the expression levels of key apoptotic regulators Bax and Bcl-2, which are upstream modulators in the classical caspase-mediated cell death cascade [39]. Typically, an increase in Bax along with a decrease in Bcl-2 promotes apoptotic progression [39, 40]. However, in BEL-7404 cells treated with MCA, protein levels of both Bax and Bcl-2 remained largely unchanged relative to untreated controls. This outcome indicates that MCA-induced cell death in these cells is unlikely to occur via the canonical Bax/Bcl-2-regulated apoptosis pathway. Consequently, alternative apoptotic mechanisms independent of Bax and Bcl-2 should be considered in explaining MCA-induced cell death.

Oxeiptosis is a ROS dependent apoptosis pathway that does not involve with Bax/Bcl-2 and their downstream apoptosis pathways [44]. DCFDA staining revealed that MCA treatment led to elevated ROS levels in both BEL-7404 and HepG2 cells in a concentration-dependent manner, with increased fluorescence observed at 2, 4, and 8 mM doses. The NAC-based ROS rescue experiment demonstrated that reactive oxygen species contribute, at least in part, to MCA-induced apoptosis in liver cancer cells. Increasing ROS production could induce degradation of KEAP1, an internal Nrf2 inhibitor [50], and release PGAM5 from Nrf2-PGAM5-AIFM1 complex [43, 44, 51]. Then, PGAM5 is translocated into mitochondria, where it dephosphorylates AIFM1. The dephosphorylated AIFM1 is the main effector to trigger oxeiptosis [51]. Western blot analysis demonstrated that exposure to 8 mM MCA for 72 h led to decreased KEAP1 expression and reduced phosphorylation of AIFM1 at Ser116 in both BEL-7404 and HepG2 cells. In parallel, qRT-PCR results indicated a significant reduction in KEAP1 mRNA levels following MCA treatment, whereas AIFM1 transcript levels remained unchanged compared to the control group. These findings support that MCA may promote apoptosis in liver cancer cells. Notably, MCA also caused a reduction in PGAM5 protein levels in both cell lines, while its mRNA levels showed no significant variation. This suggests that MCA may facilitate PGAM5 degradation at the post-translational level. However, PGAM5 downregulation has not previously been reported associated with the induction of oxeiptosis [52, 53].

While in vitro experiments confirmed the anticancer potential of MCA in BEL-7404 and HepG2 liver cancer cells, additional studies are required to validate these findings in vivo. Traditional two-dimensional (2D) monolayer cultures present significant limitations in accurately replicating the native tumor microenvironment, particularly with respect to cellular architecture and drug penetration dynamics [54]. To better simulate in vivo tumor characteristics and evaluate MCA’s therapeutic potential, a 3D multicellular tumor spheroid model was established for in vitro experiments. The results prove that MCA treatment could inhibit development of both BEL-7404 and HepG2 MCTSs. Further live and dead cell stain illustrated that more cell death occurred with incubation of 8mM, 4mM of MCA than 2mM and the negative control in both BEL-7404 and HepG2 MCTSs. The results suggest that MCA retains its efficacy in cell death induction and cell growth suppression in the BEL-7404 and HepG2 MCTS models.

The tumor xenograft nude mouse model implanted with HepG2 tumors was then used to further investigate the anticancer efficacy of MCA in an in vivo setting. A potent CDK1 inhibitor Dinaciclib was used as a positive control with 40 mg/kg dose, as described in a previous in vivo study [46]. Both 50 mg/kg and 100 mg/kg MCA treatments significantly inhibited tumor growth in HepG2 xenograft-bearing mice compared to the vehicle group after 18 days of administration. Notably, the tumor suppression observed in the 100 mg/kg MCA group was comparable to that achieved with the Dinaciclib-treated positive control. These findings confirm that MCA retains its antitumor activity in vivo. Interestingly, while Dinaciclib treatment led to a marked reduction in body weight, no significant weight loss was observed in the MCA-treated or vehicle groups, suggesting a more favorable toxicity profile for MCA. These results indicate that MCA has minor adverse effects when achieved similar tumor inhibition efficacy compared with Dinaciclib in HepG2 tumor xenograft nude mouse model.

## Conclusion

This mechanistic investigation reveals that MCA suppresses liver cancer cell growth by triggering cell cycle arrest, primarily through the suppression of CDK1 expression. Additionally, MCA promotes apoptosis via activation of the ROS-dependent oxeiptosis pathway, mediated by the NRF2–PGAM5–AIFM1 signaling axis. The subsequent in vivo study further confirmed that MCA retains its anticancer efficacy in a liver cancer xenograft mouse model while exhibiting minimal side effects. However, the precise molecular binding target of MCA remains to be identified. A deeper understanding of MCA’s mechanism of action may facilitate the design and synthesis of novel analogs with enhanced antitumor potency and improved safety profiles.

## Supplementary Information

Supplementary Material 1.

## Data Availability

No datasets were generated or analysed during the current study.

## Abbreviations

MCA: Methyl-cantharidimide
CTD: Cantharidin
HCC: Hepatocellular carcinoma
CDK1: Cyclin-dependent kinase 1
ROS: Reactive oxygen species
MCTS: Multicellular tumor spheroid
DMEM: Dulbecco’s modified eagle medium
FBS: Fetal bovine serum
DMSO: Dimethyl sulfoxide
IC₅₀: 50% inhibitory concentration
BSA: Bovine serum albumin
DAPI: 4′,6-diamidino-2-phenylindole
qRT-PCR: Quantitative reverse transcription polymerase chain reaction
RPPA: Reverse-phase protein array
PVDF: Polyvinylidene fluoride
ECL: Enhanced chemiluminescence
SDS-PAGE: Sodium dodecyl sulfate–polyacrylamide gel electrophoresis
ANOVA: Analysis of variance
GO: Gene ontology
NAC: N-acetylcysteine
AIFM: Apoptosis-inducing factor mitochondria-associated 1
PGAM5: Phosphoglycerate mutase family member 5
Nrf2: Nuclear factor erythroid 2–related factor 2
HRP: Horseradish peroxidase
TBST: Tris-buffered saline with tween 20
TBHP: Tert-Butyl hydroperoxide

## References

[1] Siegel RL, Miller KD, Wagle NS, Jemal AJC. Cancer statistics, CA Cancer J Clin. 2023. 2023;73(1):17–48. 10.3322/caac.21763 36633525

[2] Sung H, Ferlay J, Siegel RL, Laversanne M, Soerjomataram I, Jemal A, et al. Global cancer statistics 2020: GLOBOCAN estimates of incidence and mortality worldwide for 36 cancers in 185 countries. CA Cancer J Clin. 2021;71(3):209–49. 10.3322/caac.21660. 33538338 10.3322/caac.21660

[3] Center MM, Jemal A. International trends in liver cancer incidence rates. Cancer Epidemiol Biomarkers Prev. 2011;20(11):2362–8. 10.1158/1055-9965.EPI-11-0643. 21921256 10.1158/1055-9965.EPI-11-0643

[4] Kondili LA, Lazarus JV, Jepsen P, Murray F, Schattenberg JM, Korenjak M, et al. Inequities in primary liver cancer in europe: the state of play. J Hepatol. 2024;80(4):645–60. 10.1016/j.jhep.2023.12.031. 38237866 10.1016/j.jhep.2023.12.031

[5] Ye J, Gao X, Huang X, Huang S, Zeng D, Luo W et al. Integrating single-cell and spatial transcriptomics to uncover and elucidate GP73-mediated pro-angiogenic regulatory networks in hepatocellular carcinoma. Research (Wash DC). 2024;7:0387. 10.34133/research.0387 10.34133/research.0387 PMC11208919 38939041

[6] Pawlik TM, Reyes DK, Cosgrove D, Kamel IR, Bhagat N, Geschwind JF. Phase II trial of Sorafenib combined with concurrent transarterial chemoembolization with drug-eluting beads for hepatocellular carcinoma. J Clin Oncol. 2011;29(30):3960–7. 10.1200/JCO.2011.37.1021. 21911714 10.1200/JCO.2011.37.1021 PMC4829081

[7] Su J, Liu X, Zhao X, Ma H, Jiang Y, Wang X, et al. Curcumin inhibits the growth of hepatocellular carcinoma via the MARCH1-mediated modulation of JAK2/STAT3 signaling. Recent Pat Anticancer Drug Discov. 2025;20(2):145–57. 10.2174/0115748928261490231124055059. 38243928 10.2174/0115748928261490231124055059

[8] Cao LQ, Xie Y, Fleishman JS, Liu X, Chen ZS. Hepatocellular carcinoma and lipid metabolism: novel targets and therapeutic strategies. Cancer Lett. 2024;597:217061. 10.1016/j.canlet.2024.217061. 38876384 10.1016/j.canlet.2024.217061

[9] El-Serag HB, Marrero JA, Rudolph L, Reddy KR. Diagnosis and treatment of hepatocellular carcinoma. Gastroenterology. 2008;134(6):1752–63. 10.1053/j.gastro.2008.02.090. 18471552 10.1053/j.gastro.2008.02.090

[10] Ge M, Chen X-Y, Huang P, Fleishman JS, Yang D-H, Wu Z-X, et al. Understanding and overcoming multidrug resistance in cancer. Nat Reviews Clin Oncol. 2025;22(10):760–80. 10.1038/s41571-025-01059-1. 10.1038/s41571-025-01059-1 40731166

[11] Zhao H, Ling Y, He J, Dong J, Mo Q, Wang Y, et al. Potential targets and therapeutics for cancer stem cell-based therapy against drug resistance in hepatocellular carcinoma. Drug Resist Updat. 2024;74:101084. 10.1016/j.drup.2024.101084. 38640592 10.1016/j.drup.2024.101084

[12] Shi CJ, Pang FX, Lei YH, Deng LQ, Pan FZ, Liang ZQ, et al. 5-methylcytosine methylation of MALAT1 promotes resistance to Sorafenib in hepatocellular carcinoma through ELAVL1/SLC7A11-mediated ferroptosis. Drug Resist Updat. 2025;78:101181. 10.1016/j.drup.2024.101181. 39657434 10.1016/j.drup.2024.101181

[13] Jiang X, Lan Y, Zhang Y, Dong Y, Song T. LncRNA FAM83H-AS1 contributes to the Radio-resistance and proliferation in liver cancer through stability FAM83H protein. Recent Pat Anticancer Drug Discov. 2024;19(3):316–27. 10.2174/1574892818666230427164227. 37132310 10.2174/1574892818666230427164227

[14] Nakatani T, Konishi T, Miyahara K, Noda NJC. Three novel Cantharidin-Related compounds from the Chinese blister Beetle, mylabris phalerata P ALL. Bulletin. 2004;52(7):807–9. 10.1248/cpb.52.807 15256700

[15] Liu D, Chen Z. The effects of Cantharidin and Cantharidin derivates on tumour cells. Anticancer Agents Med Chem. 2009;9(4):392–6. 10.2174/1871520610909040392. 19442040 10.2174/1871520610909040392

[16] Yeh CB, Su CJ, Hwang JM, Chou MC. Therapeutic effects of Cantharidin analogues without bridging ether oxygen on human hepatocellular carcinoma cells. Eur J Med Chem. 2010;45(9):3981–5. 10.1016/j.ejmech.2010.05.053. 20691337 10.1016/j.ejmech.2010.05.053

[17] Cho WC. Evidence-based anticancer materia medica. Springer; 2011.

[18] Liang F, Wang MY, Huang WB, Li AJ. Effect of sodium cantharidinate on the angiogenesis of nude mice with human gastric cancer. Zhong Yao Cai. 2011;34(3):343–6. 21823448

[19] Shao H, Hong G, Luo X. Evaluation of sodium cantharidinate/vitamin B6 in the treatment of primary liver cancer. J Cancer Res Ther. 2014;10(1):75–8. 10.4103/0973-1482.139770. 25207897 10.4103/0973-1482.139770

[20] Deng Y-Y, Zhang W, Li N-P, Lei X-P, Gong X-Y, Zhang D-M, et al. Cantharidin derivatives from the medicinal insect mylabris phalerata. Tetrahedron. 2017;73(40):5932–9. 10.1016/j.tet.2017.08.034.

[21] Hsia TC, Lin JH, Hsu SC, Tang NY, Lu HF, Wu SH, et al. Cantharidin induces DNA damage and inhibits DNA repair-associated protein levels in NCI-H460 human lung cancer cells. Environ Toxicol. 2015;30(10):1135–43. 10.1002/tox.21986. 24639390 10.1002/tox.21986

[22] Kuo JH, Shih TY, Lin JP, Lai KC, Lin ML, Yang MD, et al. Cantharidin induces DNA damage and inhibits DNA repair-associated protein expressions in TSGH8301 human bladder cancer cell. Anticancer Res. 2015;35(2):795–804. 25667459

[23] Su CC, Liu SH, Lee KI, Huang KT, Lu TH, Fang KM, et al. Cantharidin induces apoptosis through the Calcium/PKC-Regulated Endoplasmic reticulum stress pathway in human bladder cancer cells. Am J Chin Med. 2015;43(3):581–600. 10.1142/s0192415x15500366. 25967669 10.1142/S0192415X15500366

[24] Tian X, Zeng G, Li X, Wu Z, Wang L. Cantharidin inhibits cell proliferation and promotes apoptosis in tongue squamous cell carcinoma through suppression of miR-214 and regulation of p53 and Bcl-2/Bax. Oncol Rep. 2015;33(6):3061–8. 10.3892/or.2015.3942. 25962755 10.3892/or.2015.3942

[25] Kim A, Im M, Ma JY. Sosiho–tang ameliorates cachexia–related symptoms in mice bearing colon 26 adenocarcinoma by reducing systemic inflammation and muscle loss. Oncol Rep. 2016;35(3):1841–50. 10.3892/or.2015.4527. 26718030 10.3892/or.2015.4527

[26] Ren Y, Zhang SW, Xie ZH, Xu XM, Chen LL, Lou ZG, et al. Cantharidin induces G2/M arrest and triggers apoptosis in renal cell carcinoma. Mol Med Rep. 2016;14(6):5614–8. 10.3892/mmr.2016.5963. 27878266 10.3892/mmr.2016.5963

[27] Li CC, Yu FS, Fan MJ, Chen YY, Lien JC, Chou YC, et al. Anticancer effects of Cantharidin in A431 human skin cancer (Epidermoid carcinoma) cells in vitro and in vivo. Environ Toxicol. 2017;32(3):723–38. 10.1002/tox.22273. 27113412 10.1002/tox.22273

[28] Wang G, Dong J, Deng L. Overview of Cantharidin and its analogues. Curr Med Chem. 2018;25(17):2034–44. 10.2174/0929867324666170414165253. 28413963 10.2174/0929867324666170414165253

[29] Huang X, Xie W, Yu X, Fan C, Wang J, Cao Y, et al. Methyl-Cantharidimide inhibits growth of human hepatocellular carcinoma cells by inducing cell cycle arrest and promoting apoptosis. Front Oncol. 2019;9:1234. 10.3389/fonc.2019.01234. 31803617 10.3389/fonc.2019.01234 PMC6873211

[30] Li YD, Mao Y, Dong XD, Lei ZN, Yang Y, Lin L, et al. Methyl-Cantharidimide (MCA) has anticancer efficacy in ABCB1- and ABCG2-Overexpressing and cisplatin resistant cancer cells. Front Oncol. 2020;10:932. 10.3389/fonc.2020.00932. 32676451 10.3389/fonc.2020.00932 PMC7333678

[31] Aden DP, Fogel A, Plotkin S, Damjanov I, Knowles BB. Controlled synthesis of HBsAg in a differentiated human liver carcinoma-derived cell line. Nature. 1979;282(5739):615–6. 10.1038/282615a0. 233137 10.1038/282615a0

[32] Dong XD, Lu Q, Li YD, Cai CY, Teng QX, Lei ZN, et al. RN486, a bruton’s tyrosine kinase inhibitor, antagonizes multidrug resistance in ABCG2-overexpressing cancer cells. J Translational Intern Med. 2024;12(3):288–98. 10.2478/jtim-2024-0011. 10.2478/jtim-2024-0011 PMC11284896 39081282

[33] Zhang W, Fan YF, Cai CY, Wang JQ, Teng QX, Lei ZN, et al. Olmutinib (BI1482694/HM61713), a novel epidermal growth factor receptor tyrosine kinase Inhibitor, reverses ABCG2-Mediated multidrug resistance in cancer cells. Front Pharmacol. 2018;9:1097. 10.3389/fphar.2018.01097. 30356705 10.3389/fphar.2018.01097 PMC6189370

[34] Wang N, Zhu Y, Wang L, Dai W, Hu T, Song Z, et al. Parallel analyses by mass spectrometry (MS) and reverse phase protein array (RPPA) reveal complementary proteomic profiles in Triple-Negative breast cancer (TNBC) patient tissues and cell cultures. Proteomics. 2025;25(4):e202400107. 10.1002/pmic.202400107. 39548956 10.1002/pmic.202400107

[35] Tiwari AK, Sodani K, Dai CL, Abuznait AH, Singh S, Xiao ZJ, et al. Nilotinib potentiates anticancer drug sensitivity in murine ABCB1-, ABCG2-, and ABCC10-multidrug resistance xenograft models. Cancer Lett. 2013;328(2):307–17. 10.1016/j.canlet.2012.10.001. 23063650 10.1016/j.canlet.2012.10.001 PMC3513659

[36] Morgan DO. The cell cycle: principles of control. New Science; 2007.

[37] Castedo M, Perfettini JL, Roumier T, Kroemer G. Cyclin-dependent kinase-1: linking apoptosis to cell cycle and mitotic catastrophe. Cell Death Differ. 2002;9(12):1287–93. 10.1038/sj.cdd.4401130. 12478465 10.1038/sj.cdd.4401130

[38] Choi HJ, Zhu BT. Critical role of Cyclin B1/Cdc2 up-regulation in the induction of mitotic prometaphase arrest in human breast cancer cells treated with 2-methoxyestradiol. Biochim Biophys Acta. 2012;1823(8):1306–15. 10.1016/j.bbamcr.2012.05.003. 22580043 10.1016/j.bbamcr.2012.05.003 PMC3777406

[39] Hockenbery D, Nuñez G, Milliman C, Schreiber RD, Korsmeyer SJ. Bcl-2 is an inner mitochondrial membrane protein that blocks programmed cell death. Nature. 1990;348(6299):334–6. 10.1038/348334a0. 2250705 10.1038/348334a0

[40] Oltvai ZN, Milliman CL, Korsmeyer SJ. Bcl-2 heterodimerizes in vivo with a conserved homolog, Bax, that accelerates programmed cell death. Cell. 1993;74(4):609–19. 10.1016/0092-8674(93)90509-o. 8358790 10.1016/0092-8674(93)90509-o

[41] Aldini G, Altomare A, Baron G, Vistoli G, Carini M, Borsani L, et al. N-Acetylcysteine as an antioxidant and disulphide breaking agent: the reasons why. Free Radic Res. 2018;52(7):751–62. 10.1080/10715762.2018.1468564. 29742938 10.1080/10715762.2018.1468564

[42] Vakifahmetoglu H, Olsson M, Zhivotovsky B. Death through a tragedy: mitotic catastrophe. Cell Death Differ. 2008;15(7):1153–62. 10.1038/cdd.2008.47. 18404154 10.1038/cdd.2008.47

[43] Kesavardhana S, Kanneganti TD. Stressed-out ROS take a silent death route. Nat Immunol. 2018;19(2):103–5. 10.1038/s41590-017-0034-6. 29348507 10.1038/s41590-017-0034-6

[44] Scaturro P, Pichlmair A. Oxeiptosis-a cell death pathway to mitigate damage caused by radicals. Cell Death Differ. 2018;25(7):1191–3. 10.1038/s41418-018-0134-3. 29844568 10.1038/s41418-018-0134-3 PMC6030169

[45] Han SJ, Kwon S, Kim KS. Challenges of applying multicellular tumor spheroids in preclinical phase. Cancer Cell Int. 2021;21(1):152. 10.1186/s12935-021-01853-8. 33663530 10.1186/s12935-021-01853-8 PMC7934264

[46] Parry D, Guzi T, Shanahan F, Davis N, Prabhavalkar D, Wiswell D, et al. Dinaciclib (SCH 727965), a novel and potent cyclin-dependent kinase inhibitor. Mol Cancer Ther. 2010;9(8):2344–53. 10.1158/1535-7163.MCT-10-0324. 20663931 10.1158/1535-7163.MCT-10-0324

[47] Krek W, Nigg EA. Differential phosphorylation of vertebrate p34cdc2 kinase at the G1/S and G2/M transitions of the cell cycle: identification of major phosphorylation sites. EMBO J. 1991;10(2):305–16. 10.1002/j.1460-2075.1991.tb07951.x. 1846803 10.1002/j.1460-2075.1991.tb07951.x PMC452647

[48] Gavet O, Pines J. Progressive activation of CyclinB1-Cdk1 coordinates entry to mitosis. Dev Cell. 2010;18(4):533–43. 10.1016/j.devcel.2010.02.013. 20412769 10.1016/j.devcel.2010.02.013 PMC3325599

[49] Solomon MJ, Lee T, Kirschner MW. Role of phosphorylation in p34cdc2 activation: identification of an activating kinase. Mol Biol Cell. 1992;3(1):13–27. 10.1091/mbc.3.1.13. 1532335 10.1091/mbc.3.1.13 PMC275499

[50] Leung HW, Lau EYT, Leung CON, Lei MML, Mok EHK, San Ma VW et al. NRF2/SHH signaling cascade promotes tumor-initiating cell lineage and drug resistance in hepatocellular carcinoma. Cancer Letters. 2020;476:48–56. 10.1016/j.canlet.2020.02.008 32061952

[51] Scaturro P, Pichlmair A. Oxeiptosis: a discreet way to respond to radicals. Curr Opin Immunol. 2019;56:37–43. 10.1016/j.coi.2018.10.006. 30342374 10.1016/j.coi.2018.10.006

[52] Holze C, Michaudel C, Mackowiak C, Haas DA, Benda C, Hubel P, et al. Oxeiptosis, a ROS-induced caspase-independent apoptosis-like cell-death pathway. Nat Immunol. 2018;19(2):130–40. 10.1038/s41590-017-0013-y. 29255269 10.1038/s41590-017-0013-y PMC5786482

[53] Kang P, Chen J, Zhang W, Guo N, Yi X, Cui T, et al. Oxeiptosis: a novel pathway of melanocytes death in response to oxidative stress in vitiligo. Cell Death Discov. 2022;8(1):70. 10.1038/s41420-022-00863-3. 35177586 10.1038/s41420-022-00863-3 PMC8854565

[54] Kapałczyńska M, Kolenda T, Przybyła W, Zajączkowska M, Teresiak A, Filas V et al. 2D and 3D cell cultures – a comparison of different types of cancer cell cultures. 2018;14(4):910–9. 10.5114/aoms.2016.63743 10.5114/aoms.2016.63743 PMC6040128 30002710

