# Supplementary material for: Methyl-cantharidimide suppresses cyclin-dependent kinase 1 (CDK1) and induces oxeiptosis in liver cancer
Source: J Exp Clin Cancer Res. 2026 Mar 2;45:90. doi: 10.1186/s13046-026-03669-8 (PMC13059259; doi:10.1186/s13046-026-03669-8)
Supplement: Supplementary file 1 — Supplementary Material 1. [file 13046_2026_3669_MOESM1_ESM.docx]

**Table S1. List of primers used for qPCR analysis.**

| **Gene** | **Forward primer (5’-3’)** | **Reverse primer (5’-3’)** |
| --- | --- | --- |
| *CDK1* | TTTCTTTCGCGCTCTAGCCA | GGTAGATCCGCGCTAAAGGG |
| *Keap1* | AACCGACAACCAAGACCCC | CTGCATGGGGTTCCAGAAGAT |
| *PGAM5* | ATCTGTCACGCCAACGTCATC | CAGCAAGTGAAAGAGGTCAGG |
| *AIFM* | GGCTTCCTTGGTAGCGAACTGG | GTCCAGTTGCTGAGGTATTCGG |
| *GAPDH* | CCTCAAGATCATCAGCAATGCC | TCTAGACGGCAGGTCAGGTC |

**Table S2.** **The effect of 8 mM of MCA on the cell cycle of BEL-7404 and HepG2 cell lines**

| **cell line** |  | **Treatment** | **G0/G1(%)** | **S (%)** | **G2(%)** |
| --- | --- | --- | --- | --- | --- |
| BEL-7404 |  | Control | 35.30±0.63 | 37.3±0.69 | 27.40±2.32 |
|  |  | MCA | 17.20±1.23^*^ | 33.00±1.65 | 48.80±0.13^*^ |
| HepG2 |  | Control | 47.30±0.62 | 38.70±0.79 | 14.00±0.43 |
|  |  | MCA | 16.18±1.30^*^ | 33.02±1.09 | 50.80±2.23^*^ |

Data were collected based on three independent experiments. Data are presented as mean ± Standard Deviation. *p < 0.05.


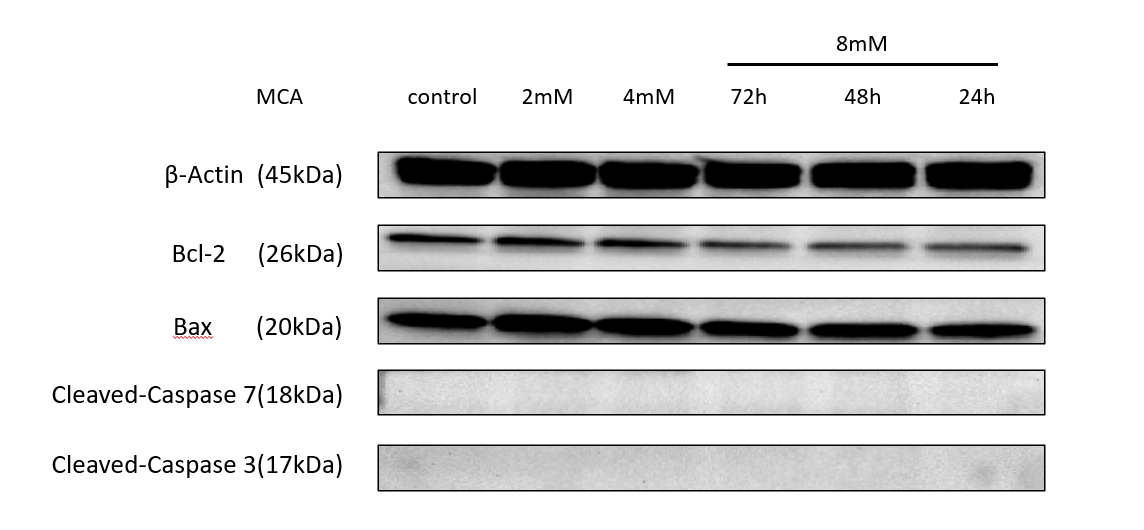


**Figure S1. Effect of MCA on the expression of apoptosis-related proteins in BEL-7404 cells.**

Western blot analysis of Bcl-2, Bax, cleaved caspase-7, and cleaved caspase-3 in BEL-7404 cells after 72-hour treatment with MCA at various concentrations (2, 4, and 8 mM). Protein expression levels were compared to the vehicle control to assess the involvement of the classical caspase-dependent apoptosis pathway.
